# The functional impairment of different subtypes and occupational states in euthymic patients with bipolar disorder

**DOI:** 10.1186/s12888-021-03242-x

**Published:** 2021-05-07

**Authors:** Xinyu Liu, Xiaojuan Ma, Wenchen Wang, Jian Zhang, Xia Sun, Xingguang Luo, Yong Zhang

**Affiliations:** 1 Henan Key Laboratory of Neurorestoratology, the first Affiliated Hospital of Xinxiang Medical University, Xinxiang, China; 2 Unit of Bipolar Disorder, Tianjin Anding Hospital, 13 Liulin Road, Hexi District, Tianjin, 300222 China; 3 Tianjin Medical College, Tianjin, China; 4 Department of Psychiatry, Yale University School of Medicine, New Haven, CT USA

**Keywords:** Bipolar disorder, Social functioning, Neurocognitive functioning, Employed, Unemployed, Subtype

## Abstract

**Background:**

The aim was to explore the associations between clinical symptoms, demographic variables, social and neurocognitive functioning in euthymic patients with bipolar disorder (BD) stratified by subgroups of DSM-IV BD (type I (BD-I) and type II (BD-II)) and occupational status (employed/unemployed), and to highlight the significance of occupational status when assessing social and neurocognitive functioning in euthymic BD patients.

**Methods:**

A total of 81 euthymic BD patients were participated in the study. The severity of the depressive and manic/hypomanic symptoms was measured using the 17-item Hamilton Depression Rating Scale (HDRS-17) and the Young Mania Rating Scale (YMRS), respectively. Social functioning and neurocognitive functioning were evaluated by the Functioning Assessment Short Test (FAST) and neurocognitive measures, respectively.

**Results:**

Employed BD patients displayed greater social functioning (autonomy, occupational functioning, interpersonal relationship domain) and better verbal learning performance and speed of processing than unemployed BD patients. The correlation between neurocognitive functioning and social functioning was stronger in the employed group than in the unemployed group. There were no significant differences in neurocognitive and social functioning between the BD-I and BD-II groups, and the correlation between neurocognitive functioning and social functioning was similar between the BD-I and BD-II groups.

**Conclusion:**

Employed BD patients may present greater occupational functioning and interpersonal relationships, as well as better verbal learning performance and speed of processing.

## Background

As a recurrent and chronic illness, bipolar disorder (BD) is a complex mental disorder characterized by pathological mood instability [1], that affects 2.4% of the global population [2]. The lifetime prevalence of bipolar disorder was 0.6% according to the China Mental Health Survey in 2012 [3]. Bipolar disorder is reported to be one of the top 20 causes of the global disease burden [4], with documented moderate to severe social and neurocognitive functional impairment [5].

Despite optimal treatment with mood stabilizers and second-generation antipsychotics, social and neurocognitive dysfunction in BD remains a serious problem. It is reported that 60–70% of BD patients have varying degrees of impairment in social and occupational functions [6]. Some findings have indicated that social and neurocognitive functional impairment persists among patients with bipolar disorder, not only at the acute stage of the illness but also in remission [7, 8]. Another research has shown that only 37.6% of the 219 BD patients achieved recovery in social functioning assessed by Global Assessment Functioning (GAF) scores after 2 years of hospitalization [9]. Social dysfunction manifests itself in a subgroup of patients even at the onset of the disease [9].

Although there are apparent social function deficits in BD patients [10], research on the differences in social function impairments between the bipolar disorder type I (BD-I) and type II (BD-II) is still inconsistent. Ruggero et al. did not find a difference in GAF scores between BD-I and BD-II patients [11]. However, Dell’Osso and his colleagues found that remitted BD and BD-I patients had significantly lower GAF scores than BD-II patients [12]. In contrast, recent studies have found differences in social function between those with BD-I and BD-II in the early stage of BD [13], suggesting that BD-II patients presented more cognitive complaints assessed by the Massachusetts General Hospital Cognitive and Physical Functioning Questionnaire and lower overall functioning when Functioning Assessment Short Test (FAST) was used to evaluate the social function. The reasons for the discrepancy in functional outcomes may be due to different assessment tools, duration of illness, education level, and pre-disease cognitive reserve [13, 14]. In terms of neurocognitive function, the prevailing view was that BD-II patients perform better than BD-I patients, but recent research has tended to support the opinion that there is no difference in neurocognitive function between the BD subtypes [15]. A meta-analysis concluded that neurocognitive differences between BD-I and BD-II are not distinct [16], which is in line with Dittmann et al.’s findings in which the Repeatable Battery for the Assessment of Neuropsychological Status (RBANS) was used to assess cognitive impairment [17].

The correlation between neurocognitive impairment and occupational status in BD patients has been mentioned in earlier studies. Altshuler et al.’s findings showed that poor executive function may lead to decreased occupational opportunities [18]. Further research suggested that working memory and speed of processing were significantly associated with occupational function in BD patients [19]. Recent findings have indicated that social function may play a core role in employment development in BD patients [20]. However, to our knowledge, there are still few studies comprehensively comparing the specific characteristics of impairment of social functioning in euthymic BD patients with different occupational status. Moreover, previous findings have been insufficient. Some functioning instrument such as GAF or the Social and Occupational Functioning Assessment Scale (SOFAS) most failed to cover all aspects of functioning in BD patients [21]. Additionally, the assessments on cognitive impairment have been also diverse, which caused inconsistence on functioning outcomes. Therefore, the efficient and multi-dimensional instruments are more likely to reflect real functional impairment in BD patients. We firstly choose the Functioning Assessment Short Test (FAST) and the MATRICS Consensus Cognitive Battery (MCCB) to evaluate the psychosocial and cognitive impairments, which could reflect real and overall functional impairments in euthymic BD patients. Our earlier findings have evidenced they had better ability of assessment to overall functioning both in BD patient and major depressive disorder patient [22, 23].

The purpose of this study was to examine the functional differences between occupational status and between subtypes in order to compare the correlation of neurocognition and social function in BD patients based on different occupational status and BD subtypes. We hypothesize that employed BD patients displayed greater scores based on the FAST assessment and better cognitive performance based on the MCCB, and euthymic BD-I patients may perform better social functioning compared to those of euthymic BD-II patients, Besides, the correlation between neurocognitive functioning and social functioning was stronger in the employed BD patients than in the unemployed BD patients.

## Methods

### Participants

Euthymic patients with bipolar disorder aged 18–60 years were recruited consecutively from Tianjin Anding Hospital between June and December 2018. Euthymic BD patients were defined as those with YMRS total score ≤ 6, HDRS-17 total score ≤ 8 and in clinical remission for at least one month [22]. Our preliminary study mainly screened BD patients who were currently euthymic, we assessed currently remitted state regardless of the number of past episodes.

Demographic information was obtained during clinical interviews. All patients met the diagnosis criteria for bipolar disorder according to the Diagnostic and Statistical Manual of Mental Disorders-fourth edition (DSM-IV) [24]. We had to exclude those patients who had manifested severe physical diseases or alcohol or substance abuse and other severe psychiatric disorders, such as schizophrenia and mental retardation. In addition, those who had received any psychological treatment, such as cognitive behavioral therapy and psychoeducation within the past six months were not included in this study. Patients who had received MECT treatment within the past six months were excluded. All recruited BD patients were permitted to use typical antipsychotic and mood stabilizer medications. We received approval from the Ethics Committee of Tianjin Anding Hospital. Written informed consent was obtained from all participants.

Occupational data was obtained as part of the clinical interview. All participants were divided into two groups based on current occupational status: employed and unemployed. Participants in the employed group consisted of individuals who were currently employed full-time (any occupational category) or enrolled as a full-time student. All participants in the unemployed group consisted of those who were currently unemployed. Participants who were retired (*n* = 1) or a “housewife” (*n* = 2) were excluded to clearly define both employed and unemployed groups [25].

### Assessments and materials

We collected sociodemographic characteristics including age, sex, education level, employment, duration of BD, family history and medication treatments in the clinical interview. The severity of the depressive and manic/hypomanic symptoms was measured using the 17-item Hamilton Depression Rating Scale (HDRS-17) [26] and the Young Mania Rating Scale (YMRS) [27], respectively. Social functional impairment was measured using the Functioning Assessment Short Test (FAST) [28]; the Chinese version of the FAST has been shown to have satisfactory reliability and validity in BD patients [22]. The FAST comprises 24 items assessing six domains of functioning: autonomy, occupational functioning, cognitive functioning, financial issues, interpersonal relationships, and leisure time [28]. The total score is the sum of all items, with a higher score indicating more severe functional impairment. The neurocognitive assessment tool was The MATRICS Consensus Cognitive Battery (MCCB), conducted by an experienced psychiatrist. The Chinese version of MCCB was translated and revised by Yu Xin’s team from the Sixth College of Peking University and established the Chinese population norm, which has excellent psychometric characteristics [29]. The speed of processing was estimated with Trail Making Test-A (TMT-A) [30] and the Symbol Digit Modalities Test (SDMT) [31]; verbal learning and visual memory were rated by the Hopkins Verbal Learning Tests-Revised (HVLT-R) and Brief Visuospatial Memory Test-Revised (BVMT-R) [32]. Executive function was evaluated by the Stroop Color-Word Test (SCWT) [33]. Color blindness was assessed in all participants before applying Stroop Color-Word Test.

### Statistical analysis

Data analysis was performed using SPSS Version 25.0 (SPSS, Chicago, IL, USA). Demographic characteristics and clinical variables were compared between the groups using *t*-tests and chi-squared tests. Furthermore, an independent samples *t*-test was conducted to compare the differences in the HDRS-17, YMRS, FAST and neurocognitive measures between groups. The correlations between occupational status, demographic risk factors, clinical symptoms, social functional impairment and neurocognitive measures were assessed by Pearson’s partial correlation coefficients. All statistical tests were two-tailed, and alpha was set at 0.05.

## Results

### Demographic characteristics for all participants

Ninety euthymic BD patients were screened in this study, and data for nine patients were excluded due to incomplete assessments. A total of 81 patients (43 male and 38 female) completed all assessments in the interview, including 45 patients with BD-I, 36 patients with BD-II, 45 employed BD patients, and 33 unemployed BD patients. No significant differences were found in demographic characteristics, including age, educational level, marital status, family history, course of BD, and number of episodes, between genders or between BD subtypes. Moreover, we did not find differences in most demographic variables, except for educational levels, between the employed and unemployed groups (see Table 1).

**Table 1:** Demographic characteristics between employed and unemployed participants.

Items	employed (*n* = 45)	unemployed (*n* = 36)	*χ2*/*t*	*p* value
	Mean (SD)	Mean (SD)		
Gender (female) (n(%))	21 (46.7%)	17 (47.2%)	0.002	0.96
Age (year)	35.3 (7.2)	32.8 (10.2)	1.30	0.22
Education levels (year)	14.0 (2.8)	11.8 (3.3)	3.23	0.002
Marital status (n(%))				
single	9 (20%)	18 (50%)		
married	34 (75.6%)	10 (27.8%)		
divorced	2 (4.4%)	7 (19.4%)		
Duration of BD (year)	7.6 (5.5)	8.6 (6.1)	−0.85	0.40
Family history (yes/no)	8/37	8/28	0.25	0.62
Number of episodes	3.4 (1.8)	3.6 (2.2)	−0.54	0.59

### Comparison of the clinical variables between the groups

We compared the clinical measures of HDRS-17, YMRS, and FAST (including its six domains), and the neurocognitive measures between genders, as well as between BD subtype groups. No significant differences in any of the above-mentioned variables were found between genders (all *p* values > 0.05). There were highly significant group differences in the FAST total scores (including the occupational functioning, cognitive functioning and interpersonal relationship domains) between BD subtype groups. BD-I group had lower scores for these variables than BD-II group, but no differences in neurocognitive measures were found between BD subtype groups (see Table 2). We also compared the clinical measures and the functional measures between the employed group and unemployed group. We found no group differences in HDRS-17 or YMRS scores (*p* > 0.05), but there were highly significant group differences in the FAST total scores, as well as in autonomy, occupational functioning, and interpersonal relationship domains, but minor differences in cognitive functioning domain. Employed patients reported lower scores in these domains than unemployed patients. In addition, the employed group reported higher scores on the Symbol Digit Modalities Test (*p* < 0.001) and Hopkins Verbal Learning Tests-Revised (*p* < 0.001) than the unemployed group. No other differences in neurocognitive measures were found between the employed group and unemployed group (see Table 2).

**Table 2:** Comparisons of clinical variables by occupational states and by BD subtypes.

Items	employed (n = 45)	unemployed (*n* = 33)	*t*	*p* value	BD-I (*n* = 45)	BD-II (*n* = 36)	*t*	*p* value
	Mean (SD)	Mean (SD)			Mean (SD)	Mean (SD)		
HDRS-17	3.7 (0.9)	3.5 (1.1)	0.96	0.338	3.6 (1.0)	3.7 (0.9)	−0.68	0.501
YMRS	3.0 (1.0)	3.5 (1.3)	−1.88	0.064	3.8 (0.9)	3.6 (1.4)	0.27	0.131
FAST total	17.5 (2.4)	25.6 (2.2)	−15.76	< 0.001	19.5 (4.0)	23.1 (4.7)	−3.81	< 0.001
autonomy	1.5 (0.5)	1.9 (0.7)	−3.07	0.003	1.6 (0.6)	1.8 (0.6)	−1.47	0.145
occupational functioning	5.2 (1.3)	9.0 (1.0)	−15.13	< 0.001	6.1 (1.8)	7.8 (2.3)	−3.64	< 0.001
cognitive functioning	2.1 (0.5)	2.4 (0.5)	−2.01	0.048	2.1 (0.5)	2.3 (0.5)	−2.05	0.044
financial issues	1.6 (0.5)	1.7 (0.5)	−0.87	0.385	1.7 (0.5)	1.6 (0.5)	0.51	0.610
interpersonal relationships	5.3 (1.2)	9.1 (1.2)	−13.61	< 0.001	6.3 (2.0)	7.9 (2.2)	−3.39	0.001
leisure time	1.8 (0.5)	1.6 (0.5)	1.08	0.286	1.7 (0.5)	1.7 (0.5)	0.15	0.879
TMT-A	38.7 (9.9)	38.6 (9.1)	0.04	0.971	39.4 (9.8)	37.8 (9.2)	0.76	0.447
SDMT	49.1 (5.7)	40.2 (8.9)	5.21	< 0.001	46.3 (8.5)	43.8 (8.5)	1.30	0.198
HVLT-R	25.6 (3.6)	19.7 (2.6)	8.17	< 0.001	23.7 (4.4)	22.1 (4.1)	1.68	0.096
BVMT-R	22.7 (4.8)	22.3 (4.8)	0.34	0.735	22.0 (4.7)	23.2 (4.7)	−1.17	0.244
SCWT (total)	179.4 (28.7)	177.3 (31.5)	0.32	0.753	177.7 (30.3)	179.4 (29.6)	−0.26	0.797

Note: BD (Bipolar Disorder); TMT-A (Trail Making Test-A); SDMT (Symbol Digit Modalities Test); HVLT-R (Hopkins Verbal Learning Tests-Revised); BVMT-R (Brief Visuospatial Memory Test-Revised); and SCWT (Stroop Color-Word Test)

### Associations between total FAST scores, neurocognitive measures and other clinical variables in stratified sampling

**Fig. 1:**
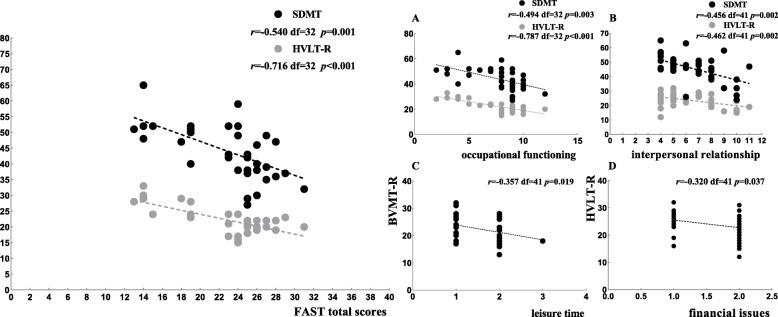
The Pearson partial correlation analysis of social function and neurocognitive measures among BD-I patients. Abbreviations: SDMT (Symbol Digit Modalities Test); HVLT-R (Hopkins Verbal Learning Tests-Revised); and BVMT-R (Brief Visuospatial Memory Test-Revised)

**Fig. 2:**
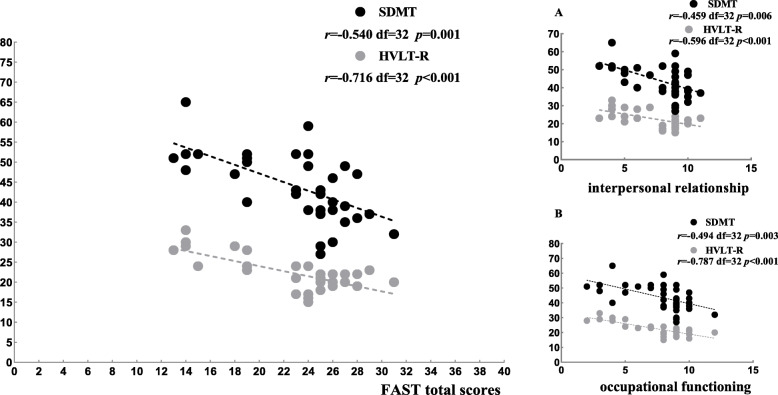
The Pearson partial correlation analysis of social function and neurocognitive measures among BD-II patients. Abbreviations: SDMT (Symbol Digit Modalities Test); HVLT-R (Hopkins Verbal Learning Tests-Revised)

We examined the correlation between FAST scores and neurocognitive measures and other variables across the BD subtypes. Our results showed that the correlations between FAST scores and neurocognitive measures were similar in the BD-I group and BD-II group. We found that FAST total scores, interpersonal relationships and occupational functioning were negatively associated with higher scores on the Symbol Digit Modalities Test and Hopkins Verbal Learning Tests-Revised in both BD subtypes. In addition, leisure time was moderately correlated with scores on the Brief Visuospatial Memory Test-Revised, and financial issues were moderately correlated with scores on the Hopkins Verbal Learning Tests-Revised in the BD-I group (See Figs. 1, 2).

**Fig. 3:**
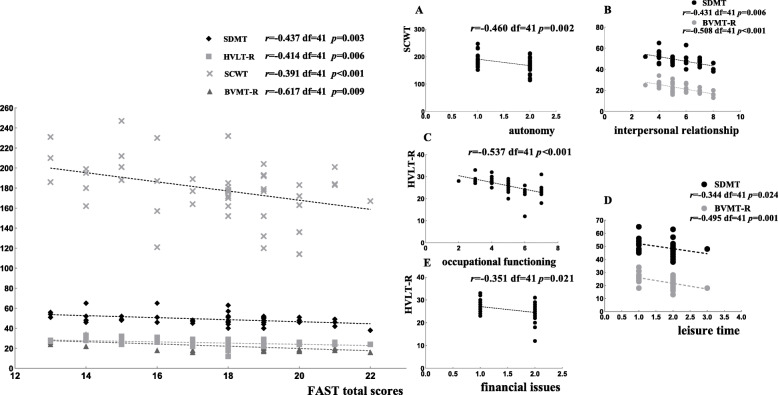
The Pearson partial correlation analysis of social function and neurocognitive measures among employed BD patients. Abbreviations: SDMT (Symbol Digit Modalities Test); HVLT-R (Hopkins Verbal Learning Tests-Revised); BVMT-R (Brief Visuospatial Memory Test-Revised); and SCWT (Stroop Color-Word Test)

We also analyzed the correlation between FAST scores and neurocognitive measures and other variables across the employed and unemployed group. As it was statistically significantly different between the employed/unemployed, we used education level as a control variable in the partial correlation analysis. In the employed group, we found that the autonomy domain was negatively related to Stroop Color-Word Test scores and occupational functioning; financial issues was negatively correlated with the Hopkins Verbal Learning Test-Revised scores and with interpersonal relationships; and leisure time was correlated with scores on the Symbol Digit Modalities Test and Brief Visuospatial Memory Test-Revised. The FAST total score was negatively associated with higher scores on the Symbol Digit Modalities Test, Hopkins Verbal Learning Test-Revised, Brief Visuospatial Memory Test-Revised and Stroop Color-Word Test (see Fig. 3).

**Fig. 4:**
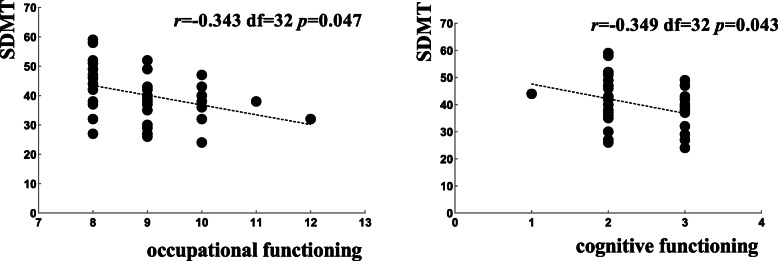
The Pearson correlation analysis of social function and neurocognitive measures among unemployed BD patients. Abbreviations: SDMT (Symbol Digit Modalities Test)

In the unemployed group, we found that only occupational functioning and cognitive functioning were related to scores on the Symbol Digit Modalities Test. No associations were found between FAST total scores or specific domains and the neurocognitive measures (see Fig. 4).

## Discussion

This preliminary study examined the associations between clinical symptoms, social function and neurocognitive function among 81 euthymic BD patients stratified by occupational status (employed/unemployed) and subgroups of DSM-IV BD (BD-I and BD-II). In line with earlier findings [34, 35], our findings showed that employed BD patients displayed greater social functioning (autonomy, occupational functioning, and interpersonal relationships) and performed better in verbal learning and processing speed than unemployed patients, indicating that employed BD patients could develop stronger social functions and some neurocognitive functions. In a previous cluster analysis study with euthymic BD patients, the group with lower functioning, measured by FAST scores, showed the highest unemployment rate, which indicated the main areas of functional loss in autonomy, occupational functioning, cognition and interpersonal relationships [36]. This is basically consistent with our findings. Some studies reported that employed BD patients showed greater neurocognitive functioning measured by the Assessment of Neuropsychological Status (RBANS), especially in the verbal memory domain [37]. Another study clearly showed that BD patients who have jobs may show better executive function than those without jobs [18], which is consistent with our results.

Further correlation analysis under stratification showed that social function outcomes had close ties with neurocognitive function among employed BD patients. There was a stronger correlation between neurocognitive function and the occupational and interpersonal relationship domains in the FAST, followed by the financial issues and autonomy domains. Verbal learning measures were predominantly associated with the occupational domain of the FAST in the employed group. Previous studies have demonstrated a strong correlation between verbal learning ability and occupational status [37], and our results confirmed this association. In addition, processing speed was also significantly correlated with the leisure time and interpersonal relationship domains in employed BD patients. we considered that employed person is more likely to participate in social activity, to facilitate self-management, and to promote learning and information-processing capacity. Many papers [16, 38–43] have reported that social functional impairment could be associated with neurocognitive measures. Remarkably the measures of processing speed, visual memory and verbal learning were powerful determinants of functional impairment in these studies, consistent with our findings for the employed group. Bearden et al. also evidenced that baseline cognitive impairment across multiple domains, particularly working memory and speed of processing, were significantly associated with concurrent occupational function impairment [19] (Bearden et al., 2011). Jaeger et al. found that baseline attention and speed of processing domains could predict functional outcomes (including occupational function) over a 12-month period [44]. Most jobs require relatively strong abilities in the areas of learning, memory and processing speed. Thus, the improvement of these functions may benefit occupational performance. Unfortunately, we found that the correlation between neurocognitive function and social function significantly weakened in the unemployed group compared with the employed group (see Figs. 3 and 4), demonstrating the poorer association between social function and neurocognition in unemployed BD patients. We presume that occupational status may be a core factor in promoting overall functional development in euthymic bipolar patients.

Our findings also testified the neurocognitive differences between the BD subtypes. There were no differences in neurocognitive measures between the BD subtypes, which is in line with earlier findings [17]. Dittmann and his colleagues evaluated psychomotor speed, working memory, verbal learning, visual / constructional abilities and executive functions in euthymic bipolar patients by using the Repeatable Battery for the Assessment of Neuropsychological Status (RBANS) [45] and the Trail Making Test (TMT) A and B [30, 46]. They also concluded that patients with both BD subtypes had similar levels of neurocognitive deficits. Similarly, another meta-analysis reported that neurocognitive differences between clinical BD subtypes were very subtle and not significant [16]. However, some studies have obtained different conclusions; for example, one study indicated that BD I patients performed worse than BD II patients in all areas of cognitive function except for working memory [47]. Another meta-analysis revealed that BD-II patients may present deficits in working memory and executive function, and more than half of the studies showed worse verbal memory. This moderate difference between BD subtypes may be complicated due to residual symptoms, number of episodes, age at illness onset, etc. [48].

Furthermore, significant differences in FAST scores and its six domains between the BD subtypes showed that BD-I patients had excellent total functioning, especially in occupational functioning and interpersonal relationships. Our findings are consistent with previous studies that showed that BD-I patients had better social function than BD-II patients did [13, 49, 50]. We considered that BD-I patients with manic episode were more likely to seek opportunities for social activity and interpersonal communication. In contrast, Dell’Osso and his colleagues found that bipolar patients with remitted BD and BD-I had significantly lower GAF scores than those with BD-II [12]. We speculate that the differences in social functioning may be influenced by functioning measures, duration of illness and education levels although no apparent differences in cognitive performance between BD subtypes [14, 51].

Besides, our correlation analyses did not suggest functional differences between the BD subtypes, indicating the same associations between processing speed and occupational functioning and interpersonal relationships both in the BD-I and BD-II patients [39]. We speculate that both BD-I and BD-II patients may exhibit similar functional impairments even in euthymia.

Our results did not show significant differences in demographic characteristics, clinical measures or social or neurocognitive functioning measures between genders. Some studies have reported that male patients could show poorer social and neurocognitive functional outcomes than female patients [41, 52], but Bücker et al. reported that males had better working memory and sustained attention than females [53]. Further study is needed to verify this gender difference in total functioning based on a larger sample size.

Several limitations of this study should be taken into consideration. First, cognitive functional assessments are scarce and do not reflect all dimensions of neurocognitive functioning in BD patients. The outcomes with no significant differences in neurocognitive measures between BD subtypes need to be tested using full-scale instruments in future research. Second, although occupational status can be viewed as an advantage of this study, current occupational status does not reflect the quality or stability of job performance, which is a particularly important area for understanding functional recovery and deserves further clarification. In addition, some studies have pointed out that the definition of occupation may influence the final assessment results [39, 54]. A prior study defined employment as including fulfilling domestic responsibilities at home or attending school [18], thus this association between total functioning and occupational status should be cautiously elucidated. Third, the possible effects of psychotropic medications on social function and neurocognitive function should be considered, and typical antipsychotics and partial mood stabilizers could hinder total functioning performance [55]. Fourth, we had no record on remission time in this study, while short remission time (at least one month) definitely weaken functioning outcomes. Finally, the sample size was not sufficient, which could reduce the statistical power when conducting comparison and correlation analysis.

## Conclusions

In summary, our findings mainly revealed that employed BD patients may present better social and neurocognitive functions, and stronger associations between social functioning and neurocognitive functioning were found in employed BD patients than in unemployed BD patients. Moreover, euthymic BD-I patients may perform better occupational functioning and interpersonal relationships compared to those of euthymic BD-II patients. However, no differences in neurocognitive impairments were found between the BD subtypes. This preliminary finding indicated that occupational status may be an important determinant that contributes to functional recovery in euthymic BD patients.

## Data Availability

The datasets used and analysed during the current study are available from the corresponding author on reasonable request.

## Abbreviations

BD: bipolar disorder
HDRS-17: 17-item Hamilton Depression Rating Scale
YMRS: Young Mania Rating Scale
FAST: Functioning Assessment Short Test
MCCB: The MATRICS Consensus Cognitive Battery
TMT-A: Trail Making Test-A
SDMT: Symbol Digit Modalities Test
HVLT-R: Hopkins Verbal Learning Tests-Revised
BVMT-R: Brief Visuospatial Memory Test-Revised
SCWT: Stroop Color-Word Test
